# The MATEX cohort – a Finnish population register birth cohort to study health effects of prenatal exposures

**DOI:** 10.1186/s12889-017-4881-8

**Published:** 2017-11-07

**Authors:** Isabell K. Rumrich, Kirsi Vähäkangas, Matti Viluksela, Mika Gissler, Heljä-Marja Surcel, Hanna de Ruyter, Jukka Jokinen, Otto Hänninen

**Affiliations:** 1 Department of Environmental and Biological Sciences, University of Eastern Finland (UEF), Kuopio, Finland; 2 Department of Public Health Solutions, National Institute for Health and Welfare (THL), Kuopio, Finland; 3 University of Eastern Finland (UEF), School of Pharmacy/Toxicology, Kuopio, Finland; 4 Department of Information Services, National Institute for Health and Welfare, Helsinki, Finland; 5 Department of Welfare, National Institute for Health and Welfare, Oulu, Finland; 6 Southern Osthrobothnia Central Hospital, Seinäjoki, Finland

**Keywords:** Register-based epidemiology, Maternal smoking, Air pollution, Finland, Developmental origin of disease

## Abstract

**Background:**

The prevalence of chronic diseases, such as immune, neurobehavioral, and metabolic disorders has increased in recent decades. According to the concept of Developmental Origin of Health and Disease (DOHaD), developmental factors associated with environmental exposures and maternal lifestyle choices may partly explain the observed increase. Register-based epidemiology is a prime tool to investigate the effects of prenatal exposures over the whole life course.

Our aim is to establish a Finnish register-based birth cohort, which can be used to investigate various (prenatal) exposures and their effects during the whole life course with first analyses focusing on maternal smoking and air pollution. In this paper we (i) review previous studies to identify knowledge gaps and overlaps available for cross-validation, (ii) lay out the MATEX study plan for register linkages, and (iii) analyse the study power of the baseline MATEX cohort for selected endpoints identified from the international literature.

**Methods/design:**

The MATEX cohort is a fully register-based cohort identified from the Finnish Medical Birth Register (MBR) (1987–2015). Information from the MBR will be linked with other Finnish health registers and the population register to link the cohort with air quality data. Epidemiological analyses will be conducted for maternal smoking and air pollution and a range of health endpoints.

**Discussion:**

The MATEX cohort consists of 1.75 million mother-child pairs with a maximum follow up time of 29 years. This makes the cohort big enough to reach sufficient statistical power to investigate rare outcomes, such as birth anomalies, childhood cancers, and sudden infant death syndrome (SIDS). The linkage between different registers allows for an extension of the scope of the cohort and a follow up from the prenatal period to decades later in life.

**Electronic supplementary material:**

The online version of this article (10.1186/s12889-017-4881-8) contains supplementary material, which is available to authorized users.

## Background

The prenatal and early postnatal period is a time of organ and tissue formation and functional programming, making these periods highly susceptible for any kind of insult. The concept of Developmental Origin of Health and Disease (DOHaD) stresses the importance of prenatal exposures for diseases later in life [1]. According to this concept, the increase in prevalence of chronic diseases (e.g. respiratory diseases, cardiovascular diseases, metabolic disorders) during the last decades is attributed to developmental aberrations associated with environmental exposures or imbalance in nutrition during pregnancy [2]. Although the DOHaD concept was originally developed with a focus on nutrition during pregnancy, it soon was applied also to toxicology and environmental health [1]. The prenatal exposure to diethylstilbestrol (DES), an artificial oestrogen and prescribed from 1940 to 1971 to millions of women during pregnancy to reduce miscarriage, is one of the earliest cases where prenatal exposure to a chemical was linked with serious health effects later in life [1]. The focus on research shifted from teratogenic effects, to effects of low-dose exposures and epigenetic changes. These epigenetic changes may explain the increase in chronic diseases, such as allergies, asthma and metabolic disorders. Most often effects of prenatal exposures are studied in animal models. Epidemiological studies on the effects of prenatal exposures are very difficult, due to the long follow-up time to catch chronic diseases which may manifest tens of years after the exposures to etiological factors.

In addition to the sufficiently long follow-up, large birth cohorts are essential to link prenatal exposures to health effects later in life, because most effects are minor or the endpoints rare, such as some birth anomalies or childhood cancers. In order to be able to detect even minor changes, which do not necessarily need to be outside the “healthy” average range, big study sizes are needed. Register based epidemiology allows to utilize large study populations and to follow them even over decades by linking various administrative registers, e.g. on health, social welfare and population. Because prenatal development is the most sensitive period of life for external insults (for reviews see e.g. [3, 4]), the possible health effects of environmental risk facts are first observable and most severe in maternally exposed offspring.

In our MATEX cohort we use the Finnish Medical Birth Register (MBR) to define our study cohort. In addition, by using the personal identification number, introduced in 1964–1968 to all Finnish citizens and permanent residents, we link our cohort with the information available in other health and population registers. Health registers have a long tradition in Finland with the earliest registers established in the 1950’s [5]. Finnish registers are regularly used in research. Between 2010 and 2016 in total 962 authorisations for data use were given by the National Institute for Health and Welfare (THL), the main holder of health registers in Finland during that period.

The MBR is frequently used in research for a wide range of topics (see Additional file 1, Chapter 3. Examples of use of MBR data in published research). Most commonly birth outcomes, such as birth weight, small for gestational age and preterm birth are used from the MBR. Other risk factors and health endpoints analysed with MBR data, include for example socioeconomic status [6, 7], maternal age [8, 9], reproductive history [10], stillbirth and neonatal mortality [11, 12], and neurodevelopmental disorders [13, 14]. The MBR data are not always used for identification of the study population or endpoints of interest, but also to identify confounding factors [15]. The maternal smoking status has been retrieved from the MBR in several studies [16–19]. Although several studies analysed birth cohorts from the MBR born between 1990 and 2010, only one birth cohort from the MBR born after 2010 has been analysed, which aimed at the maternal age at which risk for adverse pregnancy outcomes increases [20], and only one study included children born before 1990, focusing on the effect of socioeconomic status on the incidence of placental abruption [7]. No study investigated the effects of exposure to air pollution or other environmental risk factors during pregnancy on pregnancy outcomes in a Finnish birth cohort.

### Maternal smoking

Maternal smoking during pregnancy is the largest preventable factor posing a risk to the health of the mother and the child. The most severe complication associated with prenatal tobacco smoke is sudden infant death syndrome [21]. Additionally, smoking causes premature birth, low birth weight, and being small for gestational age, which all increase the risk for perinatal mortality. Nicotine, the chemical causing the addictive properties of tobacco, cause abnormal lung development in utero, which has effects throughout the life of the exposed child [22]. As a known teratogen, tobacco smoke is associated with congenital anomalies [23]. Tobacco smoke contains hundreds of chemicals, some of which are known carcinogens. In a meta-analysis it was shown that smoking during pregnancy increases the risk for brain and nervous system tumours in the childhood of the offspring [24]. Despite these well-established consequences of maternal smoking, the fraction of pregnant women smoking remained stable at around 15% in Finland from 1987 until today.

**Fig. 1:**
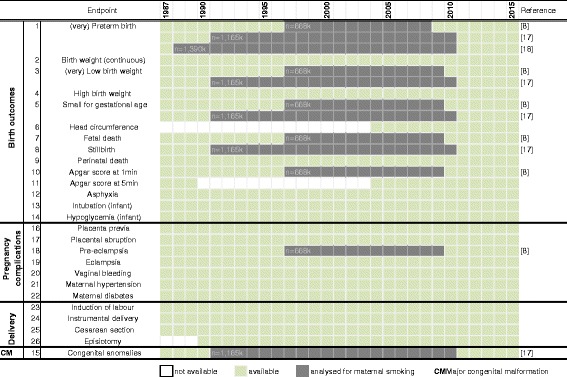
Health endpoints available in the Medical Birth Register. Annual availability of health endpoints potentially useful for the analyses of the effects of maternal smoking and air pollution. White space means that the data are not available for the year and grey marking means that the data have been analysed previously for the effects of maternal smoking

Since 1990 the smoking status of the mother is recorded in the Finnish MBR during the 1st trimester and at the end of the pregnancy, providing an excellent basis for a birth cohort focusing on the effects of maternal smoking. From 1987 to 1990 the smoking status was collected as non-smoker and below/above 10 cigarettes per day. Only a limited number of studies [8, 17, 25] have used the MBR for the analyses of the effects of maternal smoking during pregnancy on the infant (Fig. 1). These studies did not include children born after 2010 and the analyses mostly focused on low birth weight, premature birth and small for gestational age. Only one study [17] analysed major congenital malformations as part of their study. There is a clear knowledge gap in the contribution of maternal smoking to specific congenital malformations, childhood cancer and other morbidities in mother and child.

### Prenatal exposure to air pollution

The possible linkage of Finnish registers has great potential to be used in the future for the investigation of the effects of prenatal ambient air pollution on the health of infants and later in life. Ambient particulate matter (PM) pollution is by far the most significant environmental risk factor for disease and mortality both in Finland and globally. Although health effects of exposure to PM have been thoroughly studied in the adult and elderly population, the effects of prenatal exposures are not as well established. The exposure to PM_2.5_ has been associated with for example low birth weight in full term babies [26], preterm birth [27] and congenital abnormalities [28]. Only a limited number of studies analysed the effects of low concentration ambient PM (<10 μg/m^3^) [27]. The ambient PM concentration in Finland is well below the annual WHO limit of 10 mg/m^3^ for PM_2.5_. The population weighted 3-year mean (2011–2013) based on nine (sub)urban monitoring stations was 6.6 μg/m^3^ of PM_2.5_ [29]. There is a knowledge gap of the impact of prenatal exposure to low level ambient air pollution, especially for health endpoints other than low birth weight and premature birth. The Finnish MBR has not yet been used to study the effects of low level air pollution exposure during pregnancy on pregnancy outcomes and the health of the offspring later in life.

## Study design and methods

### Aims/objectives

The overall objectives of the MATEX project is the development of a framework to link a birth cohort identified from the Finnish Medical Birth Register with information from other (health) registers for analyses of prenatal exposures and their related health outcomes over the whole life course. The register-based design will allow for lifelong follow-up for the identification of critical exposure time windows and health effects, which become only apparent in later adulthood.

In the initial proof-of-concept analyses we will look at the effects of prenatal exposure to maternal smoking and probably to some extent to air pollution on pregnancy outcomes, congenital anomalies, childhood cancer, and childhood asthma. In this paper we (i) conduct a review of previous studies to identify knowledge gaps and overlaps available for cross-validation, (ii) lay out the MATEX study plan for register linkages, and (iii) analyse the study power of the baseline MATEX cohort for selected endpoints identified from the international literature.

### The MATEX birth cohort

The MATEX birth cohort is identified from the Finnish Medical Birth Register (MBR) [30]. The baseline MATEX birth cohort consists of all births recorded in the MBR between 1st January 1987 and 31st December 2015. The MATEX cohort includes personal information of the mother, such as occupation, citizenship, marital status and municipality of residence. Additionally, information on past reproductive history is available: number of previous ectopic pregnancies, miscarriages induced abortions and whether she had a Caesarean section before. The main birth outcomes are recorded: gestational age at birth, birth weight, birth length, head circumference, 1 and 5 min Apgar score, sex of the child and whether the child is born alive or dead. In addition, diagnoses are available as ICD codes (1987–1995 as ICD-9 and 1996–2015 as ICD-10), as well as information on risk factors, such as smoking and gestational diabetes or hypertension, related to pregnancy and medical treatments, e.g. drugs during pregnancy. The risk factors and treatments, which are not recorded as ICD codes are recorded as binary variable (yes/no). The sub-dataset about delivery characteristics includes only binary and categorical information for confounding analyses. Amongst other, it includes the place of birth (hospital, on the way to hospital, home), the mode of delivery, induction of labour, and procedures associated with delivery. Some of the variables have been introduced to the MBR between 1990 and 2004. See Additional file 1 for a detailed overview of the available variables (see 161 Additional file 1, Chapter 1. Data availability in the Medical Birth Register).

The MATEX baseline cohort encompassed 1,745,980 children born in a 29 year period (1987–2015), resulting in about 27.5 million person-years. The average annual number of births during that period was around 60,000 children. The average birth rate in the Finnish population was 1.2%. The majority of mothers in the MATEX cohort were Finnish, multiparous, and married or in a registered partnership. The average maternal age was 29 years (25th–75th percentile: 26–33 years). Children were on average born at 39 + 5 weeks + days gestation with a birth weight of 3.5 kg and a head circumference of 35 cm. The majority if children had an Apgar score of 9 or 10 at 5 min. Almost all deliveries were in hospital with a majority being a spontaneous vaginal birth (Table 1). For 1.2 million mothers the socioeconomic classification based on occupation is available. Around one third (34%) of all mothers was lower level employees with administrative and clerical occupations. Together less than a third of the mothers are either upper level employees with administrative, managerial, professional and related occupations (14%) or manual worker (14%). The remaining mothers are students (8%), self-employed (2%) or pensioners and un-employed (1%).

**Table 1:** Characteristics of mother-child pairs of the MATEX cohort

Characteristic	Available years*	*n* (%) or mean (25th–75th percentile)
Mother		
Age [years]		29 (26–33)
Pre-pregnancy weight [kg]	2004–2015	67 (57–73)
Height [cm]	2004–2015)	166 (161–170)
Primiparous		711,208 (41%)
Citizenship	1990–2015	
Other than Finnish		64,730 (5%)
Self-reported smoking	1990–2015	
No		1,436,322 (84%)
Quitted during 1st trimester		68,344 (4%)
Continued during 1st trimester		198,125(11%)
Marital status		
Married/registered partnership or cohabiting		1,112,771 (67%)
New-born		
Sex (Female)		853,401 (49%)
Gestational age [week + days]		39 + 5 (39 + 0–40 + 6)
Birth weight [kg]		3.5 (3.2–3.9)
Head circumference [cm]	2004–2015	35 (34–36)
Apgar score at 5 min	1987–1989, 2004–2015	
0–6		17,768 (2%)
7–8		72,137 (10%)
9–10		666,492 (88%)
Stillborn		6638 (0.4%)
Infant mortality (0–364 days of age; excluding stillbirths)		6410 (0.4%)
Number of foetuses		
Singletons		1,696,181 (97%)
Multiples		49,797 (3%)
Delivery		
Place of birth	1990–2015	
On the way to hospital		1225 (0.1%)
Outside hospital (planned)		332 (0.02%)
Outside hospital (unplanned/no information)		1126 (0.1%)
Mode of delivery		
Vaginal		1,336,572 (77%)
Breech, vaginal birth		10,186 (1%)
Vacuum		110,361 (6%)
Planned caesarean section		110,014 (6%)
Urgent/Emergency caesarean section		174,810 (10%)

^*^Given if information is not available for the whole period (1987–2015)

A general overview of the health status of the mother-child pairs are shown in the supplement in the form of incidence of chosen diseases occurring in the perinatal phase as well as congenital anomalies (see Additional file 1: Chapter 3).

### Design of register linkages

**Fig. 2:**
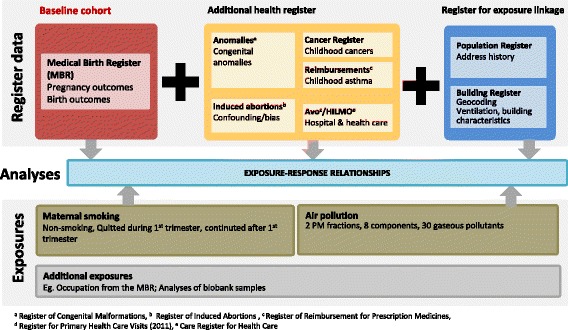
Schematic description of the planned register linkage of the MATEX cohort. The Matex cohort is recruited from the MBR with other registers for the two proof-of-concept case studies. Registers are linked via mother’s personal identification number (PIN) (for the Population Register) or the child’s PIN (all other registers). The main exposures targeted will be maternal smoking and air pollution. The main endpoints will be birth outcomes, childhood asthma, childhood cancer and congenital anomalies

The baseline MATEX cohort from the MBR will be supplemented with data from other health registers, as well as an exposure database, consisting of registers for exposure linkage such as the Population Register (Fig. 2, Table 2). All data will be pooled for analyses of exposure response relationships.

**Table 2:** The Finnish registers and their data content used in the MATEX project

Register	Data available	Register holder	Ref.
Medical Birth Register	Mother’s health data (incl. Smoking habits prior to and during pregnancy, other risk factors, interventions), pregnancy, delivery, live and stillbirths, GD, BMI, infant health data by age of 7 days	THL	[30]
Register of Induced Abortions	Data on mother, indication for induced abortion, diagnoses	THL	[38]
Register of Congenital Malformations	Mother’s health data, pregnancy, foetus/child, congenital anomalies and birth defects, diagnoses (ICD codes)	THL	[31]
Care Register for Health Care	Data from hospitals and special health care, diagnoses	THL	[33]
Register of Primary Health Care Visits	Data outpatient health care visits, diagnoses (available since 2011)	THL	[34]
Cancer Register	Persons with cancer, data on cancer type and death certificate	THL	[32]
Cause-of-Death Register	Causes of death, death certificates	Statistics Finland	[36]
Register of reimbursements for Prescription Medicines	Purchases of prescription medication eligible for reimbursement	KELA^a^	[35]
Finnish Maternity Cohort	Serum bank, data on mother, sampling date, expected due date, previous pregnancies	THL^b^	[42]
Population Register	Address history	Population Register Center	[37]

^a^Finnish Social Security Institution; ^b^From June 2017 the samples will be transferred to the Biobank Borealis of Northern Finland, established and maintained by the Northern Ostrobothnia Hospital District, the University of Oulu, NordLab and the hospital/healthcare districts of Lapland, Länsi-Pohja, Central Ostrobothnia and Kainuu

The linkage of various registers via the personal identification number (PIN) of the child enables the follow-up of the health history of the children after birth. Linkage with the Register of Congenital Malformations [31] and the Cancer Register [32] helps to identify all children of the baseline cohort, who were born with congenital anomalies, birth defects or who develop cancer later in life. The Care Register for Health Care (HILMO) [33], the Register of Primary Health Care Visits (AvoHILMO) [34] (nationwide coverage since 2011), and the Register of Reimbursement for Prescription Medicines [35] allows to identify cases of specific disorders or diseases later in life, for example asthma. The Cause-of-Death Register facilitates the possibility to study mortality associated with prenatal exposures [36]. Possible linkage with the Population Register [37] can provide the home address history of each mother-child pair, which will be used to link the cohort with air quality data for the investigation of effects of prenatal exposure to air pollution. The register-based design potentially allows for lifelong follow-up for the identification of critical exposure time windows and health effects, which become only apparent in later adulthood.

**Fig. 3:**
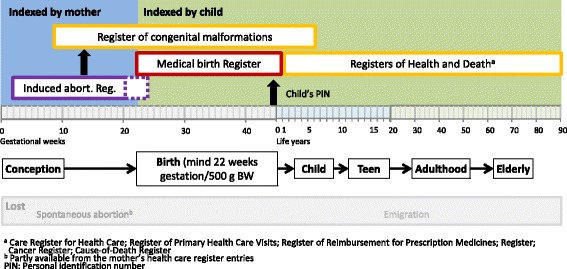
Possibility for life-long follow-up of the health status of the birth cohort by utilizing registers. The cohort can be followed using the personal identification number (PIN) of the child, except of the case of induced abortion (recorded under mother’s PIN). In the case of spontaneous abortion the record is partly available from the woman’s health register. Emigration outside of Finland causes the loss for follow up of the child

Different health registers can be used to follow the health status of the children throughout life (Fig. 3). The MBR is the main information source for the health of mother and child during pregnancy and up to the first week of life. In cases of induced abortion, no information is available in the MBR, but under the women’s PIN in the Register for Induced Abortions [38]. Induced abortions for social reasons are legally allowed until gestational week 20 and for medical reasons until gestational week 24. All live births and stillbirth from week 22 or with a minimum foetal weight of 500 g are recorded in the MBR, no matter if the foetus is stillborn or alive. Pregnancy-terminations at weeks 22–24 weeks are not to be reported to the MBR. Pregnancy loss before this milestone is legally considered to be a spontaneous abortion and the event is recorded under the women’s PIN in HILMO/AvoHilmo. A live born child receives his/her PIN at birth.

### Statistical power of the MATEX cohort

The statistical power of the MATEX cohort was estimated using R software epiR package. The smallest detectable relative risk (RR) was computed for an analyses using 95% confidence interval and a study power of 90%. The smallest detectable RR was calculated for a range of incidence rates and exposure rates for the total MATEX cohort, as well as the part of the cohort, born 2004 or later, when the latest variables were introduced to the MBR. Additionally, the smallest detectable RR was compared with published RR for outcomes associated with maternal smoking.

**Fig. 4:**
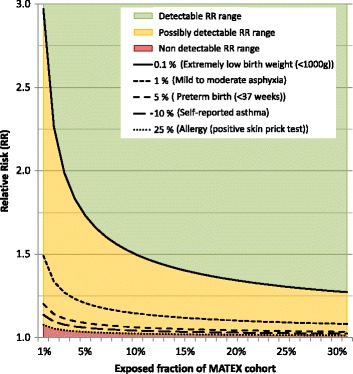
Minimum detectable RR for risk factors (RR ≥1) for five incidence rates. The minimum detectable relative risk (RR) has been estimated for five incidence rates (0.1%, 1%, 5%, 10%, 25%) and a range of an exposed fraction of the MATEX cohort between 1 and 30%. The red area describes RRs not detectable within reasonable exposure prevalence and disease incidence, the yellow area describes RRs possibly detectable depending on exposure prevalence and/or disease incidence, and the green area describes the RRs which can be detected in whole range of exposure prevalence and disease incidence. A study power of 90% has been assumed

For incidence rates between 0.1% to 50% the MATEX cohort has reasonable power (Fig. 4) to detect outcomes associated with a wide range of exposures (exposure prevalence 1% to 30%). The total MATEX cohort has 90% power to detect outcomes with an incidence rate of 0.1% and a RR ≤ 1.5 for exposure prevalent in 10% or more of the MATEX cohort.

**Fig. 5:**
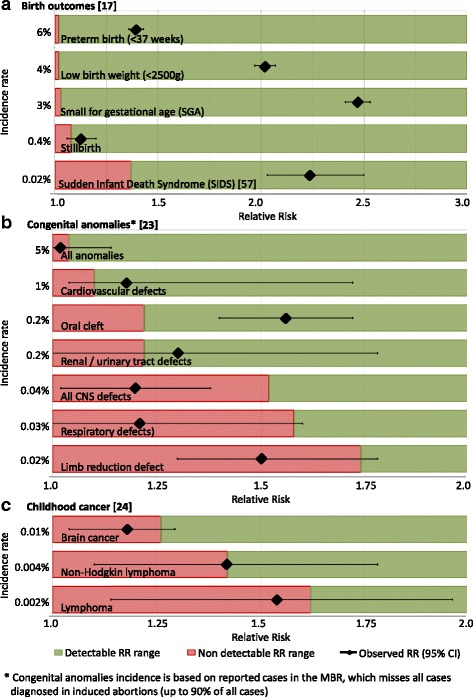
Example for the study power of the MATEX cohort and maternal smoking as exposure. The smallest detectable RR in the MATEX cohort in (study power = 90%) is compared with RR, which have been reported for birth outcomes (Panel **a**), congenital anomalies (Panel **b**) and childhood cancer (Panel **c**) [23, 57]

When planning the MATEX cohort, health endpoints associated with maternal smoking (birth outcomes, congenital anomalies, childhood cancer) have been selected based on a literature review and the needed cohort size was estimated [39]. After receiving the data, the chance to detect the reported RRs in the MATEX cohort, has been re-calculated (Fig. 5). Despite the large study population, it seems unlikely that the cohort has sufficient power to result in statistically significant risk estimates for most congenital anomalies and childhood cancers. The cohort has enough statistical power that statistically significant risk estimates can be expected for the main birth outcomes.

### Planned analyses

#### Exposures

Information on maternal smoking is retrieved from the MBR. The information is collected by the midwife during the antenatal visits. Maternal smoking status is available as four categories: (i) non-smoker, (ii) quitted smoking during first trimester, (iii) continued smoking after the first trimester, or (iv) no information. A preliminary analysis of the smoking status in the MATEX birth cohort suggests that about 15% of all children are exposed to maternal smoking at some point of the pregnancy without any trend during the study period (1987 to 2015). However, the fraction of women quitting smoking during the first trimester is increasing during the last decades. Additionally, smoking prevalence is higher in mothers younger than 20 years compared to mothers older than 30 years. In a study comparing the MBR smoking information with serum samples analysed for cotinine, the non-disclosure rate of smoking was reported to be 8% and the rate of inactive smoking mothers was reported to be 5% [40].

Air pollution exposure can be linked with the mother-child pairs via the home address of the mother during pregnancy. Modelled ambient concentrations data will be available for PM with a diameter of less than 2.5 μm (PM_2.5_) and less than 10 μm (PM_10_), as well as 30 gaseous components of air pollution. The gaseous components include amongst others carbon dioxide (CO_2_), carbon monoxide (CO), ozone (O_3_), nitrogen dioxide (NO_2_) and sulphur dioxide (SO_2_). The effect of exposure during the whole pregnancy, the three trimesters separately, and the last week of pregnancy can potentially be analysed.

The analyses can be widened by the inclusion of other exposures. The occupational status of the mothers is available as Classification of Occupation [41] and can be analysed for occupation specific risks. It is well established, that some occupations are characterised by specific exposures for examples farmers and pesticides, hair dressers and chemicals, and painters and solvents. The Finnish Maternity Cohort serum samples can be used to assess the exposure to chemicals, such as perfluorinated compounds and organochlorine pesticides [42].

#### Health outcomes

The health endpoints of interest have been selected a priori based on a literature review of previous studies. Four main categories of health endpoints will be included in the analyses: (i) pregnancy outcomes, (ii) congenital malformations, (iii) childhood cancer, and (iv) asthma.

As pregnancy endpoints mainly gestational age (preterm birth), birth weight, size for gestational age (small for gestational age, SGA), head circumference and Apgar score will be analysed. The effects of maternal smoking or air pollution on stillbirth and Sudden Infant Death Syndrome (SIDS) will be analysed. Additional analyses will potentially be done for respiratory problems in the infant (diagnoses, need for intubation) and gestational illnesses in the mother (diabetes, hypertension, (pre-)eclampsia).

The effects of prenatal exposures on congenital anomalies will be analysed for all major malformations as a group and specific malformations if the incidence rate is high enough for sufficient study power. For minimum estimates if the incidence rates, see Additional file 1: Table S2. Congenital malformation analyses will include physical anomalies as well as chromosomal and genetic anomalies. Down syndrome is of particular interest as a chromosomal anomaly.

Childhood cancer will be included as an endpoint in the analyses if sufficient study power can be reached. Leukaemia is most promising in terms of study power, because it is the most common cancer in childhood. However, the evidence for an association with maternal smoking is limited [24]. The evidence for an association of maternal smoking with brain cancer is stronger, but the incidence rate is lower, limiting the statistical power of the MATEX cohort. It is not expected that enough specific cancer cases are recorded during the study period to confidently expect sufficient study power.

Asthma can be interpreted as a continuum of respiratory problems after birth. Potentially asthma can be defined based on the entitlement for medical reimbursement for asthma medication. Additionally, data are available for diagnoses made in in- or outpatient care from the Care Register of Health Care and the Register of Primary Health Care Visits.

#### Confounding analyses

Due to the full register-based design of the MATEX study, only information recorded in the (health) register can be used for adjustment for confounding factors. In general, the assumption is that the risk for adverse pregnancy outcomes increases with a maternal age of 35 years. However, for the Finnish population it was reported, that the risk for some pregnancy outcomes increases significantly already in earlier age [20]. This underlines the importance of maternal age as a confounding factor as a continuous variable, and not as a binary variable of younger or older than 35 years.

Socio-economic status is a strong confounding factor. We will use the Finnish Classification of Socioeconomic Groups based on the occupation of the mother recorded in the MBR. The classification is based on eight main socioeconomic groups and was developed in 1989 [43]. The information is available in the MBR since 1990. Socioeconomic status is a strong indicator of health behaviour and dietary habits. Several studies showed that the risk for adverse pregnancy outcomes differs between socioeconomic groups in Finland [6, 7, 25].

Maternal smoking, gestational age, birth weight and diseases during pregnancy are available from the MBR and will be used as confounding factors as appropriate. Gestational age and (not well managed) maternal diabetes heavily affect birth weight. The child’s sex, the mother’s citizenship and birth order are available from the MBR for confounding analyses.

The effect of the physical location on the exposure-response relationship can potentially be investigated based on the municipality of residence of the mother during pregnancy. It can be used to assign the degree of urbanisation in the municipality of residence of the mother.

The Finnish health care system divides hospitals into three levels, based on the level of specialised treatment that is offered. Birth in smaller hospitals and less specialised hospitals (level 2 compared to level 3) has been shown to increase the risk for neonatal mortality [11, 12]. Information on the hospital and where each birth in the cohort was given is available and can be used to adjust for the confounding effect of hospital type.

The register-based design limits the ability to adjust for those confounding factors, which are not recorded in the registers. Paternal smoking, for example, is not recorded in any register and therefore cannot be included in any of the analyses. Information on other lifestyle factors (physical activity, diet, alcohol consumption) is not available either. Additionally, some factors, which are important for confounding analyses, such as maternal height and weight, have been introduced to the MBR only in 2004. These limitations need to be taken into account when planning for the various analyses.

#### Statistical models and possibilities for various analyses

Due to the various analyses that will be conducted, a detailed description of each of the analyses is not possible within the scope of this paper. However, mainly bi-variable analyses will be performed to evaluate statistical differences in variables between exposure groups using the χ^2^ test. For continuous variables – as applicable - independent sample t test will be used. Multivariable logistic regression analysis will be performed to calculate RRs or ORs with 95% CIs between exposure and each adverse perinatal outcome. Depending on the outcome, different statistical methods need to be used. Each analysis will be adjusted to confounding factors as appropriate. The statistical analyses will be done in R statistical software.

The rich dataset allows for analyses of continuous variables (birth weight, gestational age, head circumference) either as a continuous variable or as a categorical variable based on international disease definitions, such as (very) low birth weight and (very) preterm birth. The other variables can only be analysed as categorical or binary variables.

To some extent critical windows of exposure can be investigated. The effects of maternal smoking throughout the whole pregnancy can be compared to the effects of smoking only during the first trimester. The effects of prenatal air pollution can be analysed for average concentration or peak concentration, as well as during different periods of the pregnancy.

In order to estimate the disease burden associated with maternal smoking and air pollution, the Burden of Disease (BoD) will be estimated according to the WHO method [44]. The quantification of the health impact will provide valuable estimates about the magnitude of the health impact, as well as the severity of health endpoint in terms of healthy life lost.

## Discussion

Register-based birth cohorts provide ultimately possibilities to study the effects of prenatal and childhood exposures throughout the life course and to identify sensitive or critical time windows as is already clear from the studies carried out using the Finnish Birth Register [17, 19, 20]. In the current work our special focus is on the early postnatal period with the lifelong follow-up remaining as a readily available option. The linkage of multiple health registers supports comprehensive case identification and follow up. The current baseline cohort size of 1.75 million children leads to high study power, which is sufficient to study even rare endpoints, such as birth malformations and childhood cancer.

### Scientific knowledge gaps and potential of the MATEX birth cohort

Maternal smoking is an established cause for low birth weight and preterm birth [21]. Nevertheless, there is controversy about the association between maternal smoking and childhood cancers or specific congenital anomalies. These outcomes have low incidence rates requiring big studies to reach sufficient study power. Case-control design is often applied to identify most cases without the need for the big size of a cohort study [45]. Case-control design is retrospective leading potentially to recall bias of exposures during the pregnancy, which may have occurred decades earlier. Additionally, both case-control and cohort designs may be biased in the recruitment of the controls or study population. Register-based approach minimises the potential for both biases [5]. The health registers cover virtually the whole population. Therefore there is no risk that some population groups are over- or under-represented. The use of exposure databases minimises the risk for recall bias.

The fully register-based design limited to the available data, which, in the case of the Finnish Birth Register, have been collected for use over the 29 year recruitment period. It is should be noted that some important confounding variables are missing for certain years, such as maternal height and weight, as well as the socioeconomic group. Additionally, some possibly important confounding factors are not available, for example paternal smoking, alcohol consumption during pregnancy and physical activity. Furthermore, nicotine replacement therapy is potentially important, in the light of the fetotoxicity of nicotine found in animal studies (for a recent review, see [46]). However, it has been used only for a short period and the data has not been systematically collected in the birth register. Overall, except of maternal smoking, no lifestyle information is available. Due to the large cohort size, it is not feasible to collect additional data via questionnaires or interviews. For variables that are temporarily restricted, the cohort can be analysed in two groups (one with adjustment for the variable, one without the adjustment) to investigate the magnitude of the confounding.

Harmful effects of prenatal exposure to cigarette smoke on the 2nd generation include implications of e.g. germ cell mutations in the case of maternal smoking during pregnancy or paternal preconceptional smoking [47], but are not well-established, while the effects of air pollution have not been studied at all. The MATEX cohort is recruited over a long enough time that we can identify potential pregnancies of women, who are included in the cohort at birth. The oldest members of the cohort are currently 29 years old and the mean maternal age at pregnancy is 29 years. Later on the current focus on maternal smoking and air pollution may be widened to other prenatal exposures. The Finnish Maternity Cohort, that contains first trimester serum samples from 2 million pregnant women since 1983 (national coverage 95% of all pregnancies), as well as other Finnish blood and serum banks can be used for exposure assessment to chemicals [42]. The availability of address history has the potential to investigate exposures emitted from stationary sources, such as (nuclear) power plants, industrially contaminated sites, high voltage power lines or transformer stations (extremely low magnetic fields).

Nordic health registers are to a great extent similar, which opens the possibility for Nordic collaboration to increase the cohort size even further [48]. This would increase the study power in order to investigate rare outcomes associated with low prevalence exposures, such as illegal drugs.

Register-based epidemiology is restricted to data, which are routinely collected in registers. Most registers, however, have not been designed for research purposes per se, but rather for statistical purposes. Hence, some information important and interesting for research is missing. Information on lifestyle is not available, except of maternal smoking in the MBR. No data about paternal smoking or the use of nicotine products, such as chewing gums and skin patches, are available. Additionally, data on alcohol consumption, physical activity, eating habits and other exposures are missing. The data availability limits the possibility to adjust for confounders.

### Ethical and legal considerations

The routine collection of data for health register means more work for the health professionals, who collect the data, as well as costs to collect the data and maintain the register. These costs are paid by public funding. Utilizing data that is collected and stored anyway is cost-efficient. There is an ethical duty of the society to use the available data to improve public health and the health services. Utilization of the data not only for statistics, but also research, justifies the increased work load and costs to maintain the register. Because individuals barely have a chance to voice their opinion whether they want their data to be collected or to be used in research, the research community should do its best to make use of the data in a responsible way, not only for science, but especially to improve public and health services for the individuals. Additionally, the scientific community has the ethical responsibility to disseminate the results and conclusions both within the scientific community, and to the general public (e.g. [49]. This means that the results should not only be published in scientific journals, but also in general newspapers and responsible social media in plain language, ensuring that the public benefits, too.

Finnish and European legislation (Act on the Openness of Government Activities (621/1999) [50]; Section 8.4, Personal Data Act (523/1999) [51]; European level Directive 2016/679 (accepted 27th April 2016, to be implemented by 25 May 2018) [52] regulate the use of personal data for various purposes including research. Generally (Declaration of Helsinki [53]), and according to Finnish law (488/1999) [54] research must be based on the consent of research subjects, unless obtaining consent is unduly difficult and the research cannot be carried out without using the data. In this case the prerequisites set out in the law must be satisfied for an exception from the need for informed consent. The full register-based study design qualifies for such an exemption from the need for informed consent. Besides, the majority of Finnish public considers the benefit for public health more important than the individual right to privacy [55]. However, in the study by Eloranta and Auvinen [55], information about ongoing and new register-based research was deemed inadequate and register-based research was in general seen as an unfamiliar topic. In the MATEX study only coded information without PINs and names are being used, disabling the direct identification of individuals. Additionally, the statistical analyses do not require us to work with data of individuals, but only with the data as a set. The publication plan includes this study protocol, giving the possibility to inform the society how their data will be used. Additionally, the results of the analyses will not only be published in scientific articles, but also in newspaper articles aimed at the general public.

Data protection is crucial when health data are used, because of the importance to protect privacy and inhibit misuse of the data (e.g. [49, 56]). Individuals may be identified based on their characteristics and health history, even with unidentified data. In human biomedical studies a positive statement from an ethics committee is required by law in Finland (488/1999) before filing the request to the register holder for obtaining data. Among the crucial aspects are that data protection is sufficient and that a plan exists, what will be done with the data once the study is finished [5]. For register-based studies, no ethics committee statement is required in Finland.

## Conclusions

Register study designs provide a cost efficient opportunity to study public health impacts of environmental risk factors. In this work we establish a baseline birth cohort and demonstrate its functionality and evaluate it by studying the effects of maternal smoking. The big study size allows observations of small risks associated with common exposures. In addition, it potentially allows the inclusion of rare outcomes and rare exposures. The register-based design makes follow-up and extension of the cohort easy and straight forward. Thus, health effects of foetal exposure over the whole life course can be studied. Exposure data can be linked via home address, serum banks or additionally collected information of the mother-child pairs.

## Additional file

Additional file 1: Supplementary material. (PDF 638 kb)

## Abbreviations

AvoHILMO: Register of Primary Health Care Visits
DOHaD: Developmental Origin of Health and Disease
HILMO: Care Register for Health Care
MBR: Finnish Medical Birth Register
PIN: Personal Identification Number used in register linkages, replaced by study id in the dataset
PM_10_: Particulate matter with a diameter of ≤10 μm/m^3^
PM_2.5_: Particulate matter with a diameter of ≤2.5 μm/m^3^
RR: Relative Risk
THL: National Institute of Health and Welfare
