# Supplementary material for: The MATEX cohort – a Finnish population register birth cohort to study health effects of prenatal exposures
Source: BMC Public Health. 2017 Nov 7;17:871. doi: 10.1186/s12889-017-4881-8 (PMC5678812; doi:10.1186/s12889-017-4881-8)
Supplement: Supplementary file 1 — Supplementary material. (PDF 638 kb) [file 12889_2017_4881_MOESM1_ESM.pdf]

Supplemental material

The MATEX cohort – a Finnish population register birth cohort to study health effects of prenatal exposures

Isabell K. Rumrich [1,2,*], Kirsi Vähäkangas [3], Matti Viluksela [1,2], Mika Gissler [4], Heljä-Marja Surcel [5], Hanna de Ruyter [6], Jukka Jokinen [2], Otto Hänninen [2]

[1] University of Eastern Finland (UEF), Department of Environmental and Biological Sciences, Kuopio, Finland
[2] National Institute for Health and Welfare (THL), Department of Public Health Solutions, Kuopio, Finland
[3] University of Eastern Finland (UEF), School of Pharmacy/Toxicology, Kuopio, Finland
[4] National Institute for Health and Welfare, Department of Information Services, Helsinki Finland
[5] University of Turku
[6] Southern Ostrobothnia Central Hospital, Seinäjoki, Finland

[*] corresponding author; Isabell.Rumrich@thl.fi

Isabell K. Rumrich: Isabell.Rumrich@thl.fi
Kirsi Vähäkangas: kirsi.vahakangas@uef.fi
Matti Viluksela: matti.viluksela@thl.fi
Mika Gissler: mika.gissler@thl.fi
Heljä-Marja Surcel: helja-marja.surcel@thl.fi
Hanna de Ruyter: Hanna.deRuyter@thl.fi
Jukka Jokinen: jukka.jokinen@thl.fi
Otto Hänninen: otto.hanninen@thl.fi

Supplementary material

1. Data availability in the Medical Birth Register

The MATEX baseline cohort compromises all birth recorded in the Finnish Medical Birth Register (MBR) between 1987 and 2015. Our baseline cohort data are limited to the availability in the MBR. The MBR structure has been updated three times (1190, 1996, 2004), which affects the availability of some data (Figure S1).

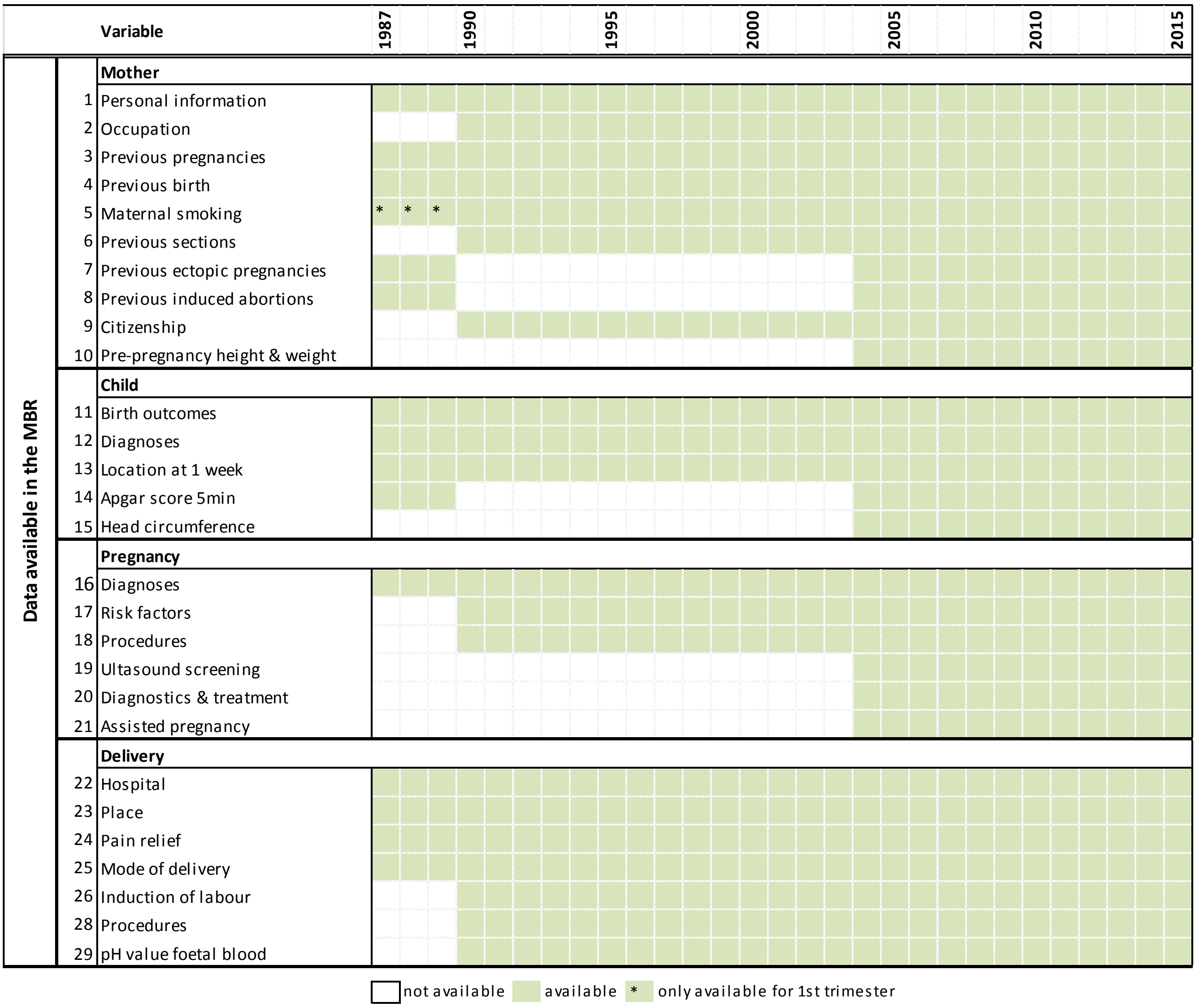

Figure S1. Example of availability of various data in the baseline MATEX cohort from the Medical Birth Register 1987-2015 (total of 1.7 million births)

Overall, roughly 100 variables are available for the analyses (Table S1). Some variables are measured continuous variables (e.g. Variables 15, 16, 37, 73, 74, 75 in Table S1), whereas other variables are categorical (e.g. Variables 3, 7, 36, 91 in Table S1) or binary (e.g. Variables 19-33, 39-65, 78-88 in Table S1). Maternal diagnoses during pregnancy, diagnoses during delivery and diagnoses in the infant until 7 day of age or discharge from hospital (whichever occurs first) are available as ICD codes (ICD-9 for 1987-1995 and ICD-10 for 1996-2015).

Table S1. Available data in the MATEX cohort

	Variable	Period available	Mean	Incidence rate[a]
1	Socioeconomic status			
2	Professional classification			
3	Municipality of Residence of mother			
4	Citizenship of mother	1990-2015		
5	Marital status of the mother			
6	Cohabiting			
7	Previous pregnancies (number)		1.5	
8	Previous miscarriages (number)		0.3	
9	Previous induced abortions (number)		0.1	
10	Previous ectopic pregnancies (number)		0.01	
11	Previous births (number)		1	
12	Previous birth when at least one infant was stillborn (number)		0.01	
13	Pre-pregnancy weight mother, kg	2004-2015	67kg	
14	Length of mother, cm	2004-2015	166cm	
15	Maternal smoking			15 %
16	Previous section	1990-2015		10 %
17	Assisted reproduction with (IVF, ICSI, FET	1990-2015		2 %
18	Intrauterine insemination	2004-2015		1 %
19	Ovulation induction	2004-2015		1 %
20	1. trimester serum screening	2004-2015		32 %
21	2. trimester serum screening	2004-2015		2 %
22	1. trimester ultrasound	1990-2015		49 %
23	2. trimester ultrasound	2004-2015		60 %
24	Chorionvillusbiopsy	1990-2015		1 %
25	Amniocentesis	1990-2015		3 %
26	Thrombosis prophylaxes	2004-2015		1 %
27	Glucoses tested	2004-2015		43 %
28	Glucoses tested and pathological	2004-2015		12 %
29	Insulin treatment started during pregnancy	2004-2015		2 %
30	Anemia (<100g/l)	2004-2015		3 %
31	Antenatal corticosteroid treatment	2004-2015		3 %
32	Maternal diagnosis during pregnancy (as ICD code)			
33	Maternity hospital			
34	Place of birth			
35	Gestational age, weeks +days, best estimate		39+5 (W+D)	
36	Mode of delivery			
37	Epidural anesthesia			30 %
38	Spinal anesthesia	2004-2015		14 %
39	Combined spinal epidural anesthesia	2004-2015		1 %
40	Paracervical analgesia	1990-2015		16 %
41	Pudendal analgesia	1990-2015		3 %
42	Nitrous oxide	1990-2015		45 %
43	Other medical pain relief	2004-2015		12 %
44	Other non-medical pain relief	2004-2015		24 %
45	Induced labor	1987-2015		17 %
46	Amniotomy	1990-2015		44 %
47	Oxytocin	1990-2015		42 %
48	Prostaglandin	1990-2015		8 %
49	Amnion infusion	2004-2015		0.1 %
50	Episiotomy	1990-2015		30 %
51	Manual extraction of the placenta	1990-2015		1 %
52	Curettage	1990-2015		1 %
53	Suturing of 3.-4. degree ruptures	2004-2015		1 %
54	Mother transferred from other hospital	1990-2015		0.3 %
55	Determining the pH value of foetal blood	1990-2015		4 %
56	Blood transfusion during birth	2003-2015		2 %
57	Placenta Previa	1990-2015		0.3 %
58	Ablatio placentae	1990-2015		0.2 %

**59**	Eclampsia	1990-2015		0.1 %
**60**	Asphyxia	1990-2015		3 %
**61**	Breech	1990-2015		3 %
**62**	Other abnormal presentation	1990-2015		3 %
**63**	Shoulder dystocia	2004-2015		0.2 %
**64**	Diagnosis at delivery (as ICD code)			
**65**	Year of birth of child	1987-2015		
**66**	Sex	1987-2015		
**67**	The infant born live/stillborn	1987-2015		0.4% [b]
**68**	Number of fetuses, 1-4	1987-2015		3 %
**69**	Code defined for each multiple birth	1987-2015		
**70**	Birth order, A-D	1987-2015		
**71**	Birth weight, g	987-2015	3.5kg	
**72**	Birth length, cm	1987-2015	50cm	
**73**	Head circumference, cm	2004-2015	35cm	
**74**	1min APGAR score	1987-2015		
**75**	5min APGAR score	1987-1990, 2004-2015		
**76**	Surveillance	1991-2015		
**77**	Treatment in other hospital	1991-2015		
**78**	Respirator treatment	1990-2015		1 %
**79**	Recovery intubation	1990-2015		1 %
**80**	Blood transfusion	1990-2015		0 %
**81**	Phototherapy	1990-2015		6 %
**82**	Antibiotics treatment	1990-2015		6 %
**83**	K vitamin treatment	2004-2015		96 %
**84**	BCG vaccination	2004-2015		22 %
**85**	Hypothyroid screening	2004-2015		95 %
**86**	Metabolic screening	2004-2015		5 %
**87**	Diagnosis of the infant (as ICD code)	1987-1990, 1996-2015		
**88**	Infant mortality	1987-2015		0.7% [c]
**89**	Perinatal mortality	1987-2015		0.6% [d]
**90**	Location of child at 7 days	1987-2015		
**91**	Maternal age at birth, years	1987-2015	29 years	
**92**	Size for gestational age	1987-2015		
**93**	Date of birth child	1987-2015		
**94**	Date of birth mother	1987-2015		
**95**	Discharge date child	1987-2015		
**96**	Date of death child	1987-2015		
**97**	Admittance date mother	1990-2015		
**98**	Discharge date mother	1990-2015		
**99**	Start last menstruation	1990-2015		

[a] for binary variables (yes/no); [b] Stillbirth rate; [c] Mortality rate during 1st year of life (including stillbirths); [d] mortality rate during 1st week of life (including stillbirths)

2. Diagnoses in the child and mother

The MATEX cohort includes any kind of diagnoses as detailed ICD codes (1987 to 1995 as ICD-9 code and 1996-2015 as ICD-10) in the mother throughout pregnancy and in the infant in the first week of life or until discharge from hospital (whichever occurred first).

For 58,090 children out of 1,167,357 a diagnoses for some kind of congenital malformation was recorded in the Medical Birth Register. The incidence rate is 5 % for any malformations. 4,313 children were diagnosed with multiple malformations. The most common type of congenital malformations are malformations and deformations of the musculoskeletal system (ICD-10 Q95-Q79), whereas congenital malformations of the nervous system (ICD-10 Q00-Q07) and of the respiratory system (ICD-10 Q30-Q34) have been recorded less than 1,000 times in 20 years (Table S2).

Table S2. Congenital malformation (according to ICD-10) incidence rate (child with at least one diagnoses per group) recorded in the Medical Birth Register between 1996 and 2015

	ICD-10 code	Incidence	Incidence rate
**Congenital malformations of the**			
**Nervous system[1]**	Q00-Q07	601	0.1 %
**Eye, ear, face and neck**	Q10-Q18	2,529	0.2 %
**Circulatory system**	Q20-Q28	11,637	1.0 %
**Respiratory system**	Q30-Q34	465	0.04 %
**Digestive system**	Q38-Q45	11,410	1.0 %
**Genital organs**	Q50-Q56	9,371	0.8 %
**Urinary system**	Q60-Q64	1,902	0.2 %
**Cleft lip and cleft palate[1]**	Q35-Q37	1,951	0.2 %
**Congenital malformations and deformations of the musculoskeletal system**	Q65-Q79	16,050	1.4 %
**Other congenital malformations[2]**	Q80-Q89	4,112	0.4 %
**Chromosomal abnormalities, not elsewhere classified[1,3]**	Q90-Q99	1,370	0.1 %

[1] Potentially heavily influenced by induced abortion leading to underestimation of true incidence in the MBR (Congenital anomalies 1993-2011, THL)
[2] for example, malformations of the skin, breast, or integument
[3] for example, Down syndrome, Edwards syndrome, Patau syndrome, Turner syndrome, Klinefelter syndrome

Children in the MATEX cohort were mainly affected by disorders related to length of gestation and foetal growth (recorded for 91,500 children). Additionally, jaundice and the effect of maternal diabetes were diagnosed regularly. The most common birth trauma was a broken collarbone. Digestive system disorders were rarely diagnosed in the first week of life. About 15,000 children were affected by asphyxia and an additional 17,500 children were affected by transient tachypnoea of new-born (Table S3).

Table S3.Selected diagnoses recorded for ICD-10 diagnoses group of "Certain conditions originating in the perinatal period" (ICD-10 codes P00-P96). Number of diagnoses are listed for the 10 disease and disorder blocks defined in ICD-10 and for each block, all specific diagnose codes are given if they were recorded at least for 1,000fetuses or newborns.[1]

	Diagnoses in Foetus and new-born	ICD-10 code	# Diagnoses	Incidence rate
**1**	**affected by maternal factors and by complications of pregnancy, labour and delivery**	**P000-P04**	**74,001**	
**1.1**	affected by maternal hypertensive disorders	P00.0	10,268	0.9 %
**1.4**	affected by premature rupture of membranes	P01.1	20,122	1.7 %
**1.6**	affected by other forms of placental separation and haemorrhage	P02.1	1,178	0.1 %
**2**	**Disorders related to length of gestation and foetal growth**	**P05-P08**	**91,509**	
**2.1**	Light for gestational age	P05.0	6,312	0.5 %
**2.2**	Small for gestational age	P05.1	10,179	0.9 %
**2.3**	Extremely low birth weight	P07.0	1,585	0.1 %
**2.4**	Other low birth weight	P07.1	20,896	1.8 %
**2.5**	Extreme immaturity	P07.2	1,898	0.2 %
**2.6**	Other preterm infants	P07.3	42,278	3.6 %
**2.7**	Exceptionally large baby	P08.0	11,875	1.0 %
**2.8**	Other heavy for gestational age infants	P08.1	5,108	0.4 %
**2.9**	Post-term infant, not heavy for gestational age	P08.2	6,905	0.6 %
**3**	**Birth trauma**	**P10-P15**	**26,912**	
**4**	**Respiratory and cardiovascular disorders specific to the perinatal period**	**P20-P29**	**68,638**	
**4.1**	Intrauterine hypoxia first noted during labour and delivery	P20.1	1,965	0.2 %
**4.2**	Severe birth asphyxia	P21.0	4,029	0.3 %
**4.3**	Mild and moderate birth asphyxia	P21.1	10,900	0.9 %
**4.4**	Respiratory distress syndrome	P22.0	6,162	0.5 %
**4.5**	Transient tachypnoea	P22.1	17,567	1.5 %
**4.6**	Other respiratory distress	P22.8	1,638	0.1 %
**4.7**	Respiratory distress, unspecified	P22.9	6,314	0.5 %
**4.8**	Neonatal aspiration of meconium	P24.0	2,131	0.2 %
**4.9**	Pneumothorax originating in the perinatal period	P25.1	2,619	0.2 %
**4.10**	Persistent foetal circulation	P29.3	2,423	0.2 %
**4.11**	Cardiovascular disorder originating in the perinatal period, unspecified	P29.9	5,757	0.5 %
**5**	**Infections specific to the perinatal period**	**P35-P39**	**35,142**	
**6**	**Haemorrhagic and haematological disorders of foetus and new-born**	**P50-P61**	**65,323**	
**6.3**	Neonatal jaundice associated with preterm delivery	P59.0	15,364	1.3 %
**6.4**	Neonatal jaundice, unspecified	P59.9	39,147	3.4 %
**7**	**Transitory endocrine and metabolic disorders specific to foetus and new-born**	**P70-P74**	**55,934**	
**7.1**	Syndrome of infant of mother with gestational diabetes	P70.0	25,274	2.2 %
**7.2**	Syndrome of infant of a diabetic mother	P70.1	7,061	0.6 %
**7.3**	Other neonatal hypoglycaemia	P70.4	25,123	2.2 %
**7.4**	Other neonatal hypocalcaemia	P71.1	1,333	0.1 %
**8**	**Digestive system disorders of foetus and new-born**	**P75-P78**	**601**	
**9**	**Conditions involving the integument and temperature regulation**	**P80-P83**	**5,488**	
**9.1**	Neonatal erythema toxicum	P83.1	1,713	0.1 %
**9.2**	Congenital hydrocele	P83.5	3,001	0.3 %
**10**	**Other disorders originating in the perinatal period**	**P90-P96**	**6,314**	

[1] Only diagnoses recorded between 1996 and 2015 are listed, because previous to 1996 ICD-9 codes were used and they are not fully compatible with the grouping of diagnoses in ICD-10.

The main complications during labour and delivery were due to malposition or malpresentation of the foetus and foetal stress. Premature rupture of membranes and false labour required maternal care most regularly. More than 70,000 mothers had diabetes mellitus during pregnancy and about 28,000 mothers developed hypertension during pregnancy (Table S4).

Table S4. Selected diagnoses in the mother related to pregnancy, childbirth and the puerperium (ICD-10 codes O00-O99). Numbers of diagnoses are listed for the 10 disease and disorder blocks defined in ICD-10 and for each block, more specific diagnoses codes are given if there were at least 1,000 times recorded.[1]

	Diagnoses	ICD-10 code	# Diagnoses	Incidence rate
**1**	**Pregnancy with abortive outcome**	**O00-O08**	**426**	
**2**	**Oedema, proteinuria and hypertensive disorders in pregnancy, childbirth and the puerperium**	**O10-O16**	**46,240**	
**2.4**	Gestational [pregnancy-induced] hypertension	O13	20,799	1.8 %
**2.5**	Pre-eclampsia	O14	14,857	1.3 %
**3**	**Other maternal disorders predominantly related to pregnancy**	**O20-O29**	**92,816**	
**3.1**	Haemorrhage in early pregnancy	O20	3,087	0.3 %
**3.3**	Venous complications and haemorrhoids in pregnancy	O22	1,223	0.1 %
**3.4**	Diabetes mellitus in pregnancy	O24	71,685	6.1 %
**4**	**Maternal care related to the foetus and amniotic cavity and possible delivery problems**	**O30-O48**	**96,056**	
**4.1**	Polyhydramnios	O40	3,503	0.3 %
**4.2**	Other disorders of amniotic fluid and membranes	O41	12,828	1.1 %
**4.3**	Premature rupture of membranes	O42	19,162	1.6 %
**4.4**	Placenta praevia	O44	3,437	0.3 %
**4.5**	Premature separation of placenta [abruptio placentae]	O45	2,014	0.2 %
**4.6**	Antepartum haemorrhage, not elsewhere classified	O46	5,177	0.4 %
**5**	**Complications of labour and delivery**	**O60-O75**	**18,4047**	
**5.1**	Preterm labour and delivery	O60	15,543	1.3 %
**5.5**	Obstructed labour due to malposition and malpresentation of foetus	O64	29,117	2.5 %
**5.6**	Obstructed labour due to maternal pelvic abnormality	O65	5,411	0.5 %
**5.9**	Labour and delivery complicated by foetal stress [distress]	O68	54,011	4.6 %
**5.10**	Labour and delivery complicated by umbilical cord complications	O69	3,996	0.3 %
**5.11**	Perineal laceration during delivery	O70	24,108	2.1 %
**6**	**Delivery**	**O80-O84**	**549,008**	
**7**	**Complications predominantly related to the puerperium**	**O85-O92**	**3,397**	
**8**	**Other obstetric conditions, not elsewhere classified**	**O94-O99**	**43,009**	

[1] Only diagnoses recorded between 1996 and 2015 are listed, because previous to 1996 ICD-9 codes were used and they are not fully compatible with the grouping of diagnoses in ICD-10.

3. Examples of use of MBR data in published research

The Finnish Medical Birth Register is used in various research fields (Table S5). Most often it is used to identify study populations for exposure outcome relationships. It is either used for identification of a cohort, or controls in case of a case-control study design. Additionally, the MBR is used to assess exposures and risk factors, for example maternal smoking and maternal age. Furthermore, the MBR has been used to study trends of disease frequency over time in the Finnish population. MBR data have been used to evaluate the quality of health care in different settings. Most studies have been published in the last ten year, but the earliest publications are from the 1990's, only a few years after the establishment of the MBR.

Table S5. Examples of previous studies utilizing the Finnish Medical Birth Register (MBR)

	Author	Title	MBR data used (years)	Exposure	Outcome under investigation
**Identification of study population for exposure-outcome study**					
**1**	[1]	Advanced maternal age, pregnancy and birth	1997-2008	Advanced maternal age (in combination smoking)	Complications during pregnancy and delivery
**2**	[2]	Maternal Smoking During Pregnancy and the Risk of Psychiatric Morbidity in Singleton Sibling Pairs	1987-1995	Maternal smoking	Psychiatric morbidity
**3**	[3]	Towards national comprehensive gestational diabetes screening - consequences for neonatal outcome 5and care	2006	Gestational diabetes	macrosomia, neonatal hypoglycaemia and the need for care in a neonatal ward
**4**	[4]	At what age does the risk for adverse maternal and infant outcomes increase? Nationwide register-based study on first births in Finland in 2005-2014	2005-2014	Maternal age	Adverse birth outcomes
**5**	[5]	Associations of maternal age with maternity care use and birth outcomes in primiparous women: a comparison of results in 1991 and 2008 in Finland	1991, 2008	Maternal age	Maternity care use, birth outcomes
**6**	[6]	Infant and childhood neurodevelopmental outcomes following prenatal exposure to selective serotonin reuptake inhibitors: overview and design of a Finnish Register-Based Study (FinESSI)	1996-2010	Selective serotonin reuptake inhibitors	Neurodevelopmental outcomes
**7**	[7]	Birth outcomes after induced abortion: a nationwide register-based study of first births in Finland	1996-2008	Previous induced abortion	Birth outcomes
**8**	[8]	The burden of OASIS increases along with socioeconomic position--register-based analysis of 980,733 births in Finland	1991-2020	Parity, socioeconomic status, smoking, maternal age, …	Obstetric anal sphincter injury

**9**	[9]	Obstetric risk factors and autism spectrum disorders in Finland	1990-2005	Obstetric factors	Autism spectrum disorders
**10**	[10]	Smoking during pregnancy and risk of autism spectrum disorder in a Finnish National Birth Cohort	1987-2005	Maternal smoking	Autism spectrum disorders
**11**	[11]	Prenatal Maternal Smoking and Tourette Syndrome: A Nationwide Register Study		Maternal smoking	Tourette syndrome
**12**	[12]	Finnish Prenatal Study of Bipolar Disorders (FIPS-B): overview, design and description of the sample	1983-1998		Bipolar disorders
**13**	[13]	Contribution of risk factors to extremely, very and moderately preterm births - register-based analysis of 1,390,742 singleton births	1987-2010	Lifestyle, reproductive risk factors	Preterm birth
**14**	[14]	Mother's education and perinatal problems in Finland	1987	Maternal education	Perinatal problems
**15**	[15]	The danger of overmatching in studies of the perinatal mortality and birthweight of infants born after assisted conception	1991-1993	Assisted pregnancy	Birth outcomes
**19**	[16]	Maternal predictors of perinatal mortality: the role of birthweight	1991-1993	Maternal risk factors	Perinatal mortality
**Studies of trends in incidence or prevalence**					
17	[17]	Eclampsia in Finland, 2006 to 2010	2006-2010	NA	Eclampsia
**18**	[18]	Decreasing mortality during pregnancy and for a year after while mortality after termination of pregnancy remains high: a population-based register study of pregnancy-associated deaths in Finland 2001–2012	2001-2012	NA	Mortality during/after pregnancy and termination of pregnancy
**19**	[19]	Fear of childbirth in nulliparous and multiparous women: a population-based analysis of all singleton births in Finland in 1997-2010	1997-2010	NA	Fear of childbirth
**20**	[20]	Social disparity affects the incidence of placental abruption among multiparous but not nulliparous women: a register-based analysis of 1,162,126 singleton births	1991-2010	NA	Placental abruption
**21**	[21]	A population-based register study to determine indications for episiotomy in Finland	1997-2007	NA	Episiotomy
**22**	[22]	Births by younger and older mothers in a population with late and regulated childbearing: Finland 1991	1991	NA	Maternal age
**Evaluation of health care quality**					

**23**	[23]	A Comparison of Medical Birth Register Outcomes between Maternity Health Clinics and Integrated Maternity and Child Health Clinics in Southwest Finland.	2009	Organisational model of clinic (maternity health clinic vs integrated maternity and child health clinic)	Utilisation of maternity care services or maternal and perinatal health outcomes
**24**	[24]	Amount of Antenatal Care Days in a Context of Effective Regionalization of Very Preterm Deliveries	2004-2006	Regionalisation of health care (Hospital level Type III vs Type II)	Antenatal care days
**25**	[25]	Effect of hospital size and on-call arrangements on intrapartum and early neonatal mortality among low-risk new-borns in Finland	2005-2009	Hospital size and on call arrangements	Early neonatal mortality (including stillbirths)
**26**	[26]	Influence of delivery characteristics and socioeconomic status on giving birth by caesarean section - a cross sectional study during 2000-2010 in Finland	2000-2010	Delivery characteristics, socioeconomic status	Delivery by caesarean section
**27**	[27]	The effect of birth in secondary- or tertiary-level hospitals in Finland on mortality in very preterm infants: a birth-register study	2000-2005	Regionalisation of health care (Hospital level Type III vs Type II)	Mortality in very preterm infants
**Evaluation of accuracy of data in the Medical Birth Register**					
**28**	[28]	Health registers as a feasible means of measuring health status in childhood--a 7-year follow-up of the 1987 Finnish birth cohort	1987	NA	Health status
**29**	[29]	Data quality after restructuring a national medical registry	1987, 1991	NA	Items recorded in the MBR

References

1. Lamminpää R: **Advanced Maternal Age, Pregnancy and Birth.** *PhD.* University of Eastern Finland, 2015

2. Ekblad M, Lehtonen L, Korkeila J, Gissler M: **Maternal Smoking During Pregnancy and the Risk of Psychiatric Morbidity in Singleton Sibling Pairs.** *Nicotine Tob Res* 2017, **19**(5):597-604.

3. Koivunen S, Torkki A, Bloigu A, Gissler M, Pouta A, Kajantie E, Vaarasmaki M: **Towards national comprehensive gestational diabetes screening - consequences for neonatal outcome and care.** *Acta Obstet Gynecol Scand* 2017, **96**(1):106-113.

4. Klemetti R, Gissler M, Sainio S, Hemminki E: **At what age does the risk for adverse maternal and infant outcomes increase? Nationwide register-based study on first births in Finland in 2005-2014.** *Acta Obstet Gynecol Scand* 2016, **95**(12):1368-1375.

5. Klemetti R, Gissler M, Sainio S, Hemminki E: **Associations of maternal age with maternity care use and birth outcomes in primiparous women: a comparison of results in 1991 and 2008 in Finland.** *BJOG* 2014, **121**(3):356-362.

6. Malm H, Artama M, Brown AS, Gissler M, Gyllenberg D, Hinkka-Yli-Salomaki S, McKeague I, Sourander A: **Infant and childhood neurodevelopmental outcomes following prenatal exposure to selective serotonin reuptake inhibitors: overview and design of a Finnish Register-Based Study (FinESSI).** *BMC Psychiatry* 2012, **12**:217-244X-12-217.

7. Klemetti R, Gissler M, Niinimaki M, Hemminki E: **Birth outcomes after induced abortion: a nationwide register-based study of first births in Finland.** *Hum Reprod* 2012, **27**(11):3315-3320.

8. Raisanen S, Cartwright R, Gissler M, Kramer MR, Heinonen S: **The burden of OASIS increases along with socioeconomic position--register-based analysis of 980,733 births in Finland.** *PLoS One* 2013, **8**(8):e73515.

9. Polo-Kantola P, Lampi KM, Hinkka-Yli-Salomaki S, Gissler M, Brown AS, Sourander A: **Obstetric risk factors and autism spectrum disorders in Finland.** *J Pediatr* 2014, **164**(2):358-365.

10. Tran PL, Lehti V, Lampi KM, Helenius H, Suominen A, Gissler M, Brown AS, Sourander A: **Smoking during pregnancy and risk of autism spectrum disorder in a Finnish National Birth Cohort.** *Paediatr Perinat Epidemiol* 2013, **27**(3):266-274.

11. Leivonen S, Chudal R, Joelsson P, Ekblad M, Suominen A, Brown AS, Gissler M, Voutilainen A, Sourander A: **Prenatal Maternal Smoking and Tourette Syndrome: A Nationwide Register Study.** *Child Psychiatry Hum Dev* 2016, **47**(1):75-82.

12. Chudal R, Sucksdorff D, Suominen A, Lehti V, Hinkka-Yli-Salomaki S, Huttunen J, Ristkari T, Gissler M, McKeague IW, Brown AS, Sourander A: **Finnish Prenatal Study of Bipolar Disorders (FIPS-B): overview, design and description of the sample.** *Nord J Psychiatry* 2014, **68**(3):169-179.

13. Raisanen S, Gissler M, Saari J, Kramer M, Heinonen S: **Contribution of risk factors to extremely, very and moderately preterm births - register-based analysis of 1,390,742 singleton births.** *PLoS One* 2013, **8**(4):e60660.

14. Hemminki E, Merilainen J, Malin M, Rahkonen O, Teperi J: **Mother's education and perinatal problems in Finland.** *Int J Epidemiol* 1992, **21**(4):720-724.

15. Gissler M, Hemminki E: **The danger of overmatching in studies of the perinatal mortality and birthweight of infants born after assisted conception.** *Eur J Obstet Gynecol Reprod Biol* 1996, **69**(2):73-75.

16. Forssas E, Gissler M, Sihvonen M, Hemminki E: **Maternal predictors of perinatal mortality: the role of birthweight.** *Int J Epidemiol* 1999, **28**(3):475-478.

17. Jaatinen N, Ekholm E: **Eclampsia in Finland; 2006 to 2010.** *Acta Obstet Gynecol Scand* 2016, **95**(7):787-792.

18. Karalis E, Ulander VM, Tapper AM, Gissler M: **Decreasing mortality during pregnancy and for a year after while mortality after termination of pregnancy remains high: a population-based register study of pregnancy-associated deaths in Finland 2001-2012.** *BJOG* 2016, .

19. Raisanen S, Lehto SM, Nielsen HS, Gissler M, Kramer MR, Heinonen S: **Fear of childbirth in nulliparous and multiparous women: a population-based analysis of all singleton births in Finland in 1997-2010.** *BJOG* 2014, **121**(8):965-970.

20. Raisanen S, Gissler M, Nielsen HS, Kramer MR, Williams MA, Heinonen S: **Social disparity affects the incidence of placental abruption among multiparous but not nulliparous women: a register-based analysis of 1,162,126 singleton births.** *Eur J Obstet Gynecol Reprod Biol* 2013, **171**(2):246-251.

21. Raisanen S, Vehvilainen-Julkunen K, Gisler M, Heinonen S: **A population-based register study to determine indications for episiotomy in Finland.** *Int J Gynaecol Obstet* 2011, **115**(1):26-30.

22. Hemminki E, Gissler M: **Births by younger and older mothers in a population with late and regulated childbearing: Finland 1991.** *Acta Obstet Gynecol Scand* 1996, **75**(1):19-27.

23. Tuominen M, Kaljonen A, Ahonen P, Makinen J, Rautava P: **A Comparison of Medical Birth Register Outcomes between Maternity Health Clinics and Integrated Maternity and Child Health Clinics in Southwest Finland.** *Int J Integr Care* 2016, **16**(3):1.

24. Helenius K, Helle E, Lehtonen L: **Amount of Antenatal Care Days in a Context of Effective Regionalization of Very Preterm Deliveries.** *J Pediatr* 2016, **169**:81-86.

25. Karalis E, Gissler M, Tapper AM, Ulander VM: **Effect of hospital size and on-call arrangements on intrapartum and early neonatal mortality among low-risk newborns in Finland.** *Eur J Obstet Gynecol Reprod Biol* 2016, **198**:116-119.

26. Raisanen S, Gissler M, Kramer MR, Heinonen S: **Influence of delivery characteristics and socioeconomic status on giving birth by caesarean section - a cross sectional study during 2000-2010 in Finland.** *BMC Pregnancy Childbirth* 2014, **14**:120-2393-14-120.

27. Rautava L, Lehtonen L, Peltola M, Korvenranta E, Korvenranta H, Linna M, Hallman M, Andersson S, Gissler M, Leipala J, Tammela O, Hakkinen U, PERFECT Preterm Infant Study Group: **The effect of birth in secondary- or tertiary-level hospitals in Finland on mortality in very preterm infants: a birth-register study.** *Pediatrics* 2007, **119**(1):e257-63.

28. Gissler M, Hemminki E, Louhiala P, Jarvelin MR: **Health registers as a feasible means of measuring health status in childhood--a 7-year follow-up of the 1987 Finnish birth cohort.** *Paediatr Perinat Epidemiol* 1998, **12**(4):437-455.

29. Gissler M, Teperi J, Hemminki E, Merilainen J: **Data quality after restructuring a national medical registry.** *Scand J Soc Med* 1995, **23**(1):75-80.
